# Diversity and Distribution of Freshwater Aerobic Anoxygenic Phototrophic Bacteria across a Wide Latitudinal Gradient

**DOI:** 10.3389/fmicb.2017.00175

**Published:** 2017-02-22

**Authors:** Isabel Ferrera, Hugo Sarmento, John C. Priscu, Amy Chiuchiolo, José M. González, Hans-Peter Grossart

**Affiliations:** ^1^Departament de Biologia Marina i Oceanografia, Institut de Ciències del Mar, Consejo Superior de Investigaciones CientíficasBarcelona, Spain; ^2^Department of Experimental Limnology, Leibniz Institute of Freshwater Ecology and Inland FisheriesStechlin, Germany; ^3^Department of Hydrobiology, Universidade Federal de São CarlosSão Carlos, Brazil; ^4^Department of Land Resources and Environmental Sciences, Montana State UniversityBozeman, MT, USA; ^5^Department of Microbiology, University of La LagunaLa Laguna, Spain; ^6^Department of Biochemistry and Biology, Potsdam UniversityPotsdam, Germany

**Keywords:** AAP bacteria, photoheterotrophy, *puf*M gene, freshwater lakes, latitudinal gradients, biogeography

## Abstract

Aerobic anoxygenic phototrophs (AAPs) have been shown to exist in numerous marine and brackish environments where they are hypothesized to play important ecological roles. Despite their potential significance, the study of freshwater AAPs is in its infancy and limited to local investigations. Here, we explore the occurrence, diversity and distribution of AAPs in lakes covering a wide latitudinal gradient: Mongolian and German lakes located in temperate regions of Eurasia, tropical Great East African lakes, and polar permanently ice-covered Antarctic lakes. Our results show a widespread distribution of AAPs in lakes with contrasting environmental conditions and confirm that this group is composed of different members of the *Alpha*- and *Betaproteobacteria*. While latitude does not seem to strongly influence AAP abundance, clear patterns of community structure and composition along geographic regions were observed as indicated by a strong macro-geographical signal in the taxonomical composition of AAPs. Overall, our results suggest that the distribution patterns of freshwater AAPs are likely driven by a combination of small-scale environmental conditions (specific of each lake and region) and large-scale geographic factors (climatic regions across a latitudinal gradient).

## Introduction

Aerobic anoxygenic phototrophs (AAPs) use organic carbon (C) for their metabolism and growth but can derive a portion of their energy requirements by harvesting light using bacteriochlorophyll *a* (BChl*a*). Their discovery last decade in the ocean challenged our view of the aquatic C cycle in which heterotrophic bacteria were considered strict consumers of organic matter produced by photosynthetic phytoplankton (Kolber et al., 2000, 2001). Intense investigations have shown that marine AAPs typically account for 1–10% of total prokaryotes in the euphotic zones of the world's oceans (Koblížek, 2015), are phylogenetically and metabolically diverse (Fuchs et al., 2007; Yutin et al., 2007; Koblížek et al., 2010, 2011; Ferrera et al., 2014) and exhibit high growth rates (Koblížek et al., 2007; Ferrera et al., 2011). Although non-marine AAPs have received less attention, they have been described in ecosystems such as rivers (Ruiz-González et al., 2013), estuaries (Waidner and Kirchman, 2007; Cottrell et al., 2010), and lakes (Karr et al., 2003; Mašín et al., 2008, 2012; Salka et al., 2011; Fauteux et al., 2015). These studies suggest that AAPs may be higher contributors to total bacterial biomass in inland waters than in marine environments. High numbers have been reported in various lakes from central Europe (up to 21%; Mašín et al., 2008, 2012), mountain lakes in Austria (up to 22%; Čuperová et al., 2013), temperate and boreal lakes of Québec (up to 37%; Fauteux et al., 2015) and acidified mountain lakes in the Czech Republic (up to 60%; Mašín et al., 2008). These reports have likewise pointed out that freshwater AAP bacterial contributions undergo large seasonal oscillations, peaking in summer or early autumn (Čuperová et al., 2013; Lew et al., 2015). Furthermore, recent evidence indicates that AAPs play relevant ecological roles in freshwater ecosystems, particularly in the C cycle by contributing disproportionately to total bacterial production (Stegman et al., 2014; Garcia-Chaves et al., 2016). However, these studies are restricted to regional studies of temperate, boreal and high-altitude freshwater biomes in the Northern hemisphere, and information on the occurrence of AAPs in other regions is lacking.

Despite the accumulating information on AAPs in inland waters, information of their phylogenetic diversity is still scarce and mostly restricted to boreal and temperate regions. The few available studies suggest that freshwater systems are dominated by different members of the *Alpha*- and *Betaproteobacteria* (e.g., Waidner and Kirchman, 2007; Cottrell et al., 2010; Salka et al., 2011; Fauteux et al., 2015). A striking difference with marine systems is that gammaproteobacterial AAPs are almost absent in freshwater environments (Caliz and Casamayor, 2014) as opposed to marine environments where they can be prevalent (Lehours et al., 2010; Ferrera et al., 2014). In particular, different members of the *Betaproteobacteria* have been found to dominate freshwater lakes in various geographic locations such as the German lakes in the Mecklenburg Lake district (*Rhodoferax*-like; Salka et al., 2011), temperate lakes in North America (*Polynucleobacter*-like; Martinez-Garcia et al., 2012) and oligotrophic high mountain lakes in Central Pyrenees, Spain (*Limnohabitan*s; Caliz and Casamayor, 2014). A thorough study in several mountain lakes in the Tyrolean Alps (Austria) showed that while the lakes under the tree-line were inhabited by a variety of both *Alpha*- and *Betaproteobacteria* AAPs, lakes located at higher altitudes were dominated by members of *Sphingomonadales* (*Alphaproteobacteria*; Čuperová et al., 2013). Trophic status, conductivity, pH, nitrate concentration and resistance to UV radiation have been proposed as drivers of the observed diversity patterns (Mašín et al., 2012; Čuperová et al., 2013; Caliz and Casamayor, 2014). Despite these reports, we still have a poor understanding of the diversity and distribution of AAPs in inland waters. Expanding the study of AAP abundance and diversity to other latitudes will improve our understanding of the factors affecting patterns of species distribution and abundance of this ecologically important bacterial group.

In this study, we investigated AAP abundance and phylogenetic diversity in freshwater ecosystems in a set of lakes from three continents, covering temperate (Mongolian and German lakes in Eurasia), tropical (Great East African lakes) and polar (Antarctic Dry Valleys lakes) regions. We assessed AAP diversity by cloning and sequencing of the *puf*M gene, and estimated *puf*M gene abundance by quantitative polymerase chain reaction (qPCR). Links between AAP abundance, diversity and an array of environmental variables were also explored to better understand the spatial dynamics of AAP communities.

## Experimental procedures

### Sampling and DNA extraction

Lakes located in four different geographic areas were sampled in this study: (1) Mongolian Lakes Gun Selenge, Terkhiin Tsagaan, Gun Arkhangai, Boon Tsagaan and Ugii Nuur; (2) African Lakes Victoria, Kivu, Edward, and Albert; (3) McMurdo Dry Valleys Antarctic Lakes Bonney, Fryxell, Hoare, Miers and Vanda; and (4) Lake Stechlin located in the Mecklenburg Lake district (Northeastern Germany; Table 1). Data from German Lakes Grosse Fuchskuhle, Haus, Roofen, Stolp and Stechlin previously published by Salka et al. (2011) were included in this study for comparison. The Antarctic campaign was conducted by the McMurdo (MCM) Dry Valleys Long-Term Ecological Research program in the Austral summer of 2011-2012. African samples were collected during the Damas-II Expedition in May 2012, and Mongolian samples were collected by Gongor Sergelen in August 2008. The sample from Lake Stechlin was collected through the Leibniz-Institute of Freshwater Ecology and Inland Fisheries monitoring program in summer 2013. All lakes were sampled between 9:00 and 12:00 h (local time) in the upper oxygenated layers of the water column at different intervals using standard methodologies. The permanently ice-covered (~4 m) Antarctic lake samples were collected through a borehole in the ice cover. Environmental data were collected at the time of sampling using standard procedures. Samples for DNA extraction were filtered onto 0.2 μm polycarbonate filters and stored at −20°C until further processing. DNA was extracted using phenol-chloroform as described previously in Salka et al. (2011) and used for the molecular analyses.

**Table 1:** **Values of variables measured in the lakes included in this study (TP, total phosphorous; SRP, soluble reactive phosphorus; TN: total nitrogen; DOC, dissolved organic carbon)**.

**Lake**	**Location Lat/Lon**	**Altitude (m)**	**Sfc area (km^2^)**	**Length (km)**	**Max width (km)**	**Mean depth (m)**	**Max depth (m)**	**Volume (km^3^)**	**T (°C)**	**pH**	**Conductivity (μS/cm)**	**TP (μg L^−1^)**	**SRP (μg L^−1^)**	**TN (mg L^−1^)**	**${\text{NH}}_{4}^{+}$ (μg L^−1^)**	**${\text{NO}}_{2}^{-}$ (μg L^−1^)**	**${\text{NO}}_{3}^{-}$ (μg L^−1^)**	**DOC (mg L^−1^)**	**Trophic state**	**Depths analyzed (m)**	**AAP Abundance (%)**
**MONGOLIA**																					
Gun Selenge	50.25;106.6	600	2.5	–	–	2	5	0.005	24	9.1	–	–	–	–	–	–	–	–	Eutrophic	0–4	1.2
Terkhiyn Tsagaan	48.17;99.72	2,060	61	18	6	6	20	0.369	13.6	8.7	–	–	–	–	–	–	–	–	Oligotrophic	0–5	2.3
Gun Arkhangai	48.41;106.89	1,431	1.7	2.4	1.1	3	7.7	0.0056	20.0	8.9	–	–	–	–	–	–	–	–	Eutrophic	0–3	17.9
Boon Tsagaan	45.32;99.97	1,312	252	24	19	9.9	16	2.385	22.0	8.9	–	–	–	–	0.15	0.02	1	–	Oligo/Mesotrophic	0–5	1.4
Ugiy Nuur	47.77;102.73	1,332	25.7	7.4	5.3	6.6	16	0.171	14.5	8.6	–	–	0.1	–	–	–	–	–	Meso/Oligotrophic	0–3	8.2
**AFRICA**																					
Victoria	− 0.55;33.27	1,134	68,800	370	350	40	84	2750	25	8.4	95	75	24.9	–	7.4	0	45.1	1.81	Meso/Eutrophic	0–5	0.6
Kivu	− 2.57;29.63	1,460	2,220	105	60	240	480	333	24.4	8.8	1,211	18	1.32	–	1.4	0	34	1.86	Oligotrophic	5	0.6
Edward	0.2;29.83	912	2,325	84	42	17	112	39.52	26.7	9	850	42	14.1	–	1.25	0.7	39.6	4.81	Oligotrophic	0–5	0.6
Albert	1.81;31.26	615	5,300	170	42	25	58	132.5	27.9	8.9	627	75	1.8	–	14.8	0.7	20.9	4.4	Oligotrophic	0–7m	2.2
**ANTARCTICA**																					
East Lake Bonney	− 77.71;162.45	57	3.32	5.2	0.9	15	37	0.05	2.00	4.25	1,504	1.6	–	–	34	–	831	1.5	Oligotrophic	4–6	0.7
West Lake Bonney	− 77.72;162.37	57	0.99	1.8	0.5	22	40	0.015	1.97	3.9	1,324	1.6	–	–	24	–	615	1.2	Oligotrophic	4–6	0.3
Fryxell	− 77.61;163.15	18	7.08	5.8	2.1	3.2	20	0.025	1.25	3.9	491	2.56	–	–	0.9	–	–	4.4	Mesotrophic	4.5–6	0.6
Hoare	− 77.65;162.85	73	1.94	4.2	1	9	34	0.018	0.60	5	339	0.64	–	–	1.26	–	163	1.9	Oligotrophic	4–6	0.8
Miers	− 78.12;163.9	170	1.3	1.5	0.7	–	21	–	1.50	4.7	–	1.6	–	–	2.5	–	–	1.1	Oligotrophic	4–6	0.2
Vanda	− 77.57;161.19	50	5.2	8	2	30.8	75	0.16	8.00	–	–	0.05	–	–	–	–	–	–	Oligotrophic	40	3.4
GERMANY^*^																					
Grosse Fuchskuhle NE	53.11;12.99	57	3.36	–	–	3.3	5.5	0.00001	22.7	6.6	ca. 30	24	5	0,95	37	7	76	25.09	Eutrophic	1	–
Grosse Fuchskuhle NW	53.11;12.99	57	3.36	–	–	3.3	5.5	0.00001	22.5	6.1	ca. 30	32	2	0,58	36	3	13	–	Eutrophic	1	–
Grosse Fuchskuhle SW	53.11;12.99	57	4.43	–	–	2.8	4.5	0.00001	21.4	4.5	ca. 40	47	1	1,25	37	0	7	65.86	Eutrophic/Dystrophic	1	4.3
Haus	53.34;13.45	84	1.31	1.91	0.86	4.9	12.5	0.00815	20.2	8.6	ca. 300	44	4	0,91	36	0	14	23.9	Eutrophic	1	3.3
Roofen	53.34;13.45	62	0.57	–	–	–	19.1	–	22.2	8.4	ca. 300	15	1	1,06	36	7	296	–	Mesotrophic	1	1.9
Stechlin	53.34;13.45	60	4.52	3.7	2.4	22.8	69	0.0969	20.4	8.7	ca.300	11	1	0,29	10	2	23	10.64	Oligotrophic	1	6.5
Stolp	53.18;13.21	52	3.71	3.6	1.41	6.64	13	0.0247	19.8	8.6	–	44	1	0,74	36	2	16	11.49	Eutrophic	1	5.8

* *Data from Salka et al. (2011)*.

### Quantitative polymerase chain reaction

Abundance of AAP and total bacteria were assessed by quantitative polymerase chain reaction (qPCR) of the *puf*M gene, a marker for AAPs, and the 16S rRNA gene, a marker for total bacteria. Primer pair pufM557F and pufM_WAWR was used for qPCR of the *puf*M gene (Waidner and Kirchman, 2008); the total bacterial primers used were 530F-N (5′-TGCCAGCMGCNGCGG-3′) modified after Garcia et al. (2011), and 926R (5′-CCGTCAATTCCTTTRAGTTT-3′; Baker et al., 2003). Gene abundances in 1 μl of DNA (10 ng μl^−1^) were measured on a CFX96 thermocycler (Bio-Rad, Berkeley, CA, USA) using Maxima SYBR Green qPCR Master Mix (2X; Fermentas, Schwerte, Germany) and the PCR conditions previously described for *puf*M (Waidner and Kirchman, 2008) and the 16S rRNA gene (Salka et al., 2014). Standard curves for *puf*M were generated from the amplification of DNA from *Limnohabitans planktonicus* and standard curves for total bacteria from *Escherichia coli* DNA kindly provided by Sarahi Garcia (Garcia et al., 2014). Relative abundance of AAPs (%AAPs) was estimated at different depths in the upper epilimnion of each lake as the percentage of total bacteria carrying the *puf*M gene assuming two copies of the 16S rRNA gene per bacterial cell and one *puf*M gene per AAP cell. The average number of 16S rRNA and *puf*M gene copies was estimated from the complete genomes belonging to AAP species that are available in GenBank (www.ncbi.nlm.nih.gov/genbank) and Ensembl databases (www.ensembl.org). Values shown here are average abundances at the different depths analyzed.

### Diversity of *puf*M

Before *puf*M clone library construction, we investigated the presence of the *puf*M gene at different depths with Denaturing Gradient Gel Electrophoresis (DGGE) as described by Salka et al. (2011). Based on DGGE results (data not shown) and physicochemical data available, we selected a range of sample depths to be further analyzed by clone libraries. Depths sampled were located in the upper part of the oxic epilimnion to prevent the amplification of anaerobic photosynthetic populations. Clone libraries were constructed from Mongolian (Gun Selenge, Terkhiin Tsagaan, Gun Arkhangai, Boon Tsagaan and Ugii Nuur), African (Victoria, Kivu, Edward, and Albert) and Antarctic (East and West lobes of Lakes Bonney, Fryxell, Hoare, Miers and Vanda) lakes, and from a sample from Lake Stechlin (Germany). Clone libraries were constructed using the same procedures as Salka et al. (2011) in order to compare our dataset to the data previously published. *puf*M gene was amplified by using forward primer pufL (5′-CTKTTCGACTTCTGGGTSGG-3′) and reverse primer pufM (5′-CCATSGTCCAGCGCCAGAA-3′) as described in Salka et al. (2011). Amplicons obtained (~1500 bp gene fragments) from different depths (see Table 1) were pooled and cloned following the procedures described in Salka et al. (2011). Approximately 50 clones from each library were sequenced by Eurofins Sequencing Services (Berlin, Germany) using the pufM primer.

### Phylogenetic analysis

The *puf*M clone sequences obtained were analyzed using the GENEIOUS software version 4.8.5 (Biomatters Limited). Chimera formations were identified by UCHIME (Edgar et al., 2011). Chimeric sequences, short sequences and sequences presenting weak signals were excluded from further analyses. Curated sequences were merged with sequences included in the previous study from German lakes (Salka et al., 2011) and grouped into operational taxonomic units (OTUs) or phylotypes using UCLUST (Edgar, 2010) with a minimum identity of 95%. A representative sequence from each phylotype was chosen by selecting the most abundant sequence within that particular phylotype and used for phylogenetic analysis. A custom database of aligned sequences was constructed in ARB (Ludwig, 2004) based on the sequences reported by Salka et al. (2011), Čuperová et al. (2013), and Caliz and Casamayor (2014) and adding additional reference sequences available in GenBank. Representative nucleotide sequences were imported and aligned in ARB. A selection of reference sequences from the custom database were chosen for the phylogenetic tree reconstruction. Later, a 50% base frequency filter was calculated including only sequence positions with more than 50% identity. This reduces the influence of highly variable sequences on the tree topology. Phylogenetic trees were constructed with RAxML (Stamatakis, 2014) under the GTRGAMMA base substitution model. The iTOL tool was used to visualize the tree output as well as the geographic and latitudinal categories (Letunic and Bork, 2007). Sequence data has been deposited in GenBank database under accession numbers LT547860- LT548047.

### Statistical analyses

Linear regressions and pairwise correlations (Pearson's correlation coefficient) were used to assess the links between AAP abundance and physicochemical variables using the JMP software (SAS Institute, Cary, NC, USA). Variables were log-transformed when necessary. The results were threshold at *P* < 0.05 and FDR-corrected for multiple comparisons (Pike, 2011). Richness (Chao1), diversity indices (Shannon) and analyses of variance (ANOVA) between multiple factors and abundance and diversity estimates were performed in R (http://www.R-project.org, R base and Vegan package). For community analyses, a dissimilarity matrix (Bray–Curtis) was constructed based on the relative abundance (square root transformed) of each phylotype. Patterns of community structure were visualized using hierarchical cluster and non-metrical multidimensional (nMDS) analyses. Mantel tests were conducted to find correlations between the phylotype matrix and a matrix of environmental variables (Euclidean distance matrix; altitude, surface area, length, max width, mean depth, max depth, volume, catchment area, conductivity, temperature, pH, total phosphorous, phosphate, ammonium, nitrite, nitrate, and dissolved organic carbon). The null hypothesis (H_o_) of “no relationship between matrices” was tested applying Spearman rank's correlation coefficient and 999 permutations. Additionally, using the ENVFIT function, we tested for significant relationships between these environmental variables and the nMDS ordination of samples. Finally, permutational tests (PERMANOVA) were employed to examine the community differences among lake trophic status, and geographic and latitudinal regions. Community statistical analyses were run in R (Vegan package; Oksanen et al., 2013).

## Results and discussion

### Lake features

The geographic and limnological characteristics of the lakes studied are summarized in Table 1. Lakes were located in four distinct geographic regions: (i) the Mongolian plateau in the arid steppe region of Northeast Asia, (ii) the tropical East African Great Lakes, (iii) the perennially ice-covered lakes of the McMurdo (MCM) Dry Valleys located in East Antarctica, the coldest and driest desert on earth, and (iv) the glacial lakes of the Mecklenburg district (Northeastern Germany; Table 1). The lakes in the Mecklenburg district were previously studied by Salka et al. (2011) using the same methodologies and the data have been included in our study. Moreover, an additional sample taken from the German Lake Stechlin was newly analyzed. This set of lakes covers a wide range of climatic and environmental conditions, particularly in temperature (ranging from 0.6 to 27.9°C), but also varying in terms of surface area, depth, pH, conductivity and trophic status, from oligotrophic to eutrophic, at local and global scales (see Table 1).

### Abundance of AAPs

The presence of AAPs was detected in all lakes surveyed confirming their widespread distribution in contrasting freshwater ecosystems including perennially ice-covered lakes. However, the percentage of bacteria carrying this gene varied widely spanning from less than ~1% to ~18%, a trend that has recurrently been observed (Mašín et al., 2008, 2012; Čuperová et al., 2013; Fauteux et al., 2015). Estimated values are within the range reported in other freshwater ecosystems (Mašín et al., 2008, 2012; Čuperová et al., 2013; Fauteux et al., 2015). AAPs have been enumerated previously either by infrared microscopy or by qPCR. Microscopy abundance estimates rely on the visualization of the BChl*a* pigment and thus, only AAP cells expressing the pigment at the moment of sampling are enumerated which may lead to their underestimation. Measures based on qPCR suffer from other biases related for example to primer specificity (Yutin et al., 2005) or to the heterogeneity of 16S rRNA gene copy number in different bacterial taxa (Crosby and Criddle, 2003). However, due to its simplicity qPCR has become a routine technique for the quantification of microbial populations, including AAPs (Waidner and Kirchman, 2008; Ritchie and Johnson, 2012). Despite these plausible methodological limitations, the numbers obtained by qPCR are well within the range determined by microscopy in other limnic systems. Furthermore, abundance of AAPs in Lake Stechlin estimated in this study by qPCR (4.3%) is in the same range than previous numbers obtained by microscopy also in summer season (5.6–13%; Mašín et al., 2012).

The relative abundance of AAPs was on average higher in temperate lakes than in tropical and Antarctic lakes. Values within each region varied considerably (see Table 1); the highest numbers were obtained from Mongolian Lakes Gun Arkhangai and Ugii Nuur, which harbored 17.9 and 8.2% of total bacteria, respectively. These values are closer to the highest numbers reported for freshwater lakes (Mašín et al., 2008, 2012; Fauteux et al., 2015). Conversely, AAP abundances were the lowest in Antarctic lakes with the exception of extremely oligotrophic Lake Vanda (3.4%). These lakes are perennially ice-covered, and low light availability (<10% of incident) may limit the development of AAPs. Interestingly, recorded temperatures were very low in all Antarctic lakes except in Lake Vanda, which has warm-saline bottom waters (Spigel and Priscu, 1998), and samples were collected at a depth where the water column had a temperature of 8°C. Nevertheless, when analyzing the entire dataset no significant correlation between temperature and global abundance data was found (*P* > 0.05). In fact, the highest temperatures (average 26°C) correspond to the great African lakes where low abundances of AAPs were found.

In seasonal surveys carried out in temperate latitudes, AAPs have been shown to increase in numbers with increasing temperature in marine (Ferrera et al., 2014) and freshwater environments (Mašín et al., 2008; Čuperová et al., 2013; Fauteux et al., 2015). Total phosphorous, Chl*a* concentration, DOC (Dissolved organic C) concentration, DOC:Chl*a* ratio, water transparency, and lake altitude have also been shown to influence AAP abundance in certain freshwater lakes (Mašín et al., 2008; Fauteux et al., 2015). As for temperature, we found no significant correlation between AAP abundance and any of the variables tested. An important difference between our study and those published previously is that we are comparing lakes from contrasting climatic regions while previous studies surveyed glacial temperate and boreal lakes of the Northern hemisphere (Québec and Northern and Central Europe) at local scales. In our study environmental conditions fail to explain the observed results probably because differences between climatic zones may be stronger.

Another factor potentially influencing the abundance of AAP bacteria is the trophic status of the lakes. Previous reports (Mašín et al., 2008, 2012) have shown that AAPs display a pattern remarkably similar to what has been consistently observed for phototrophic picoplankton; their abundance increases with increasing trophy, but their relative contribution to total phytoplankton biomass decreases with increasing trophic status (reviewed by Callieri, 2008). However, our data did not show any obvious trend between the %AAP and the trophic conditions. Thus, these results do not support a clear and consistent dependency of AAP abundance on the trophic state, at least at this global scale.

### Community structure

**Figure 1:**
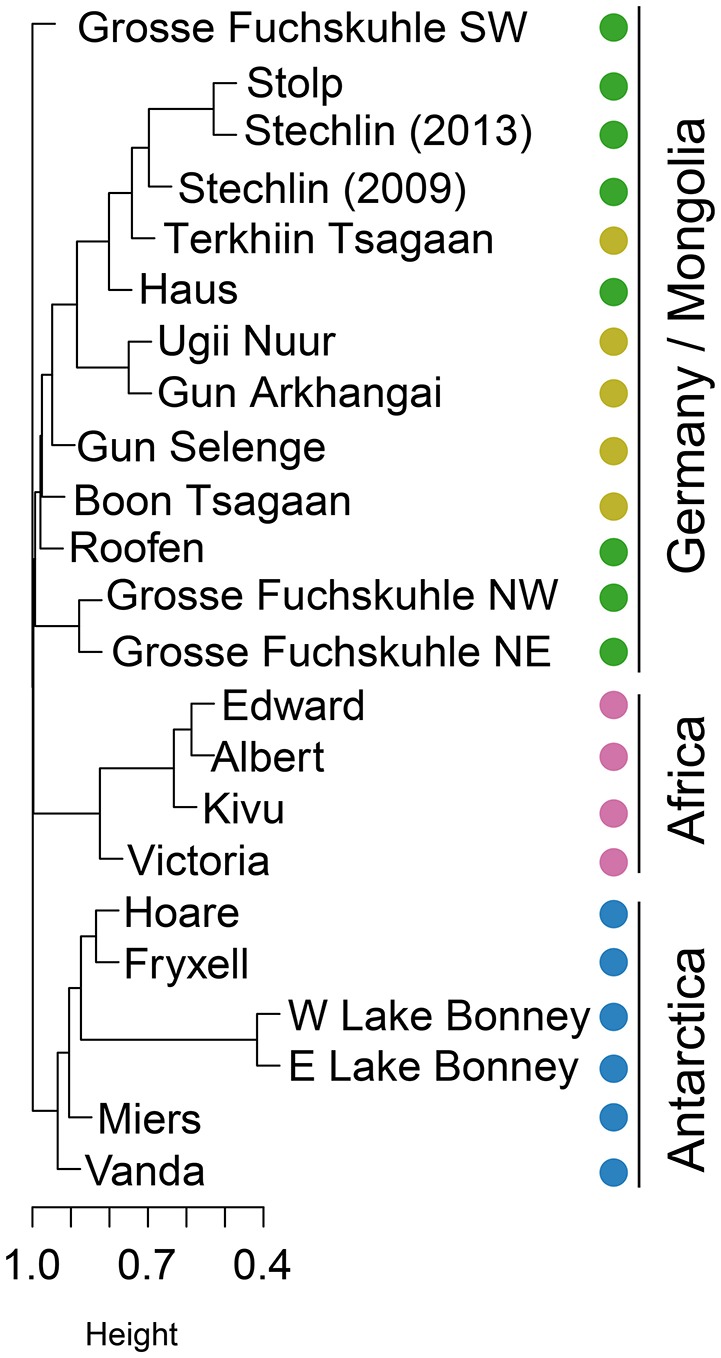
**Hierarchical clustering based on OTU relative abundance**. Clustering reflects how similar AAP assemblages are from each other based on their distance derived from the Bray-Curtis similarity coefficients calculated from the square root transformed relative abundance of each OTU. Samples are indicated by colors given their geographical location (Germany: dark green; Mongolia: light green; Africa: pink; Antarctica: blue).

As opposed to more connected environments such as rivers, estuaries, and oceans, lakes have been traditionally seen as biogeographical islands of patchy distribution even when located in the same region. Some evidences seem to support the hypothesis that bacterial distribution shows, to some extent, biogeographical patterns in these ecosystems (see reviews by Dolan, 2005; Brendan Logue and Lindström, 2008). Yet, most studies conclude that bacterial communities are controlled by a combination of geographic and environmental factors (Hanson et al., 2012). Nevertheless, recent reports revise this view of lakes as biogeographical islands and contrarily, contemplate lakes as highly connected hydrologic networks in which bacteria can be exchanged (Ruiz-González et al., 2015; Niño-García et al., 2016). We did observe clear differences in community structure among regions (beta-diversity; Figure 1). AAP assemblages from tropical and polar regions clustered according to their geographic location, but German and Mongolian clustered together despite being thousands of kilometers away (Figure 1). An exception was the SW basin of the artificially divided Lake Grosse Fuchskuhle, which represents a separated branch in the clustering (Figure 1). This basin is acidic and humic matter rich and was previously shown to have a community of AAP largely differing from other German lakes (Salka et al., 2011).

Among Antarctic lakes, the East and West lobes of Lake Bonney were more similar as expected, and these two formed a cluster with Lakes Hoare and Fryxell. These three lakes are located in Taylor Valley, whereas Lake Vanda and Lake Miers are found in separated valleys (Wright and Miers Valleys respectively). Lake Vanda was the most dissimilar lake compared to the rest of Antarctic lakes. Interestingly, Lake Vanda has unusually warm waters for such cold environments possibly due to a seawater origin (Angino and Armitage, 1963). Regarding the cluster of African lakes, Lake Victoria was the most dissimilar. Lake Victoria has a lower conductivity than Lakes Edward, Albert, and Kivu, and is a meso- to eutrophic lake compared to the other oligotrophic African lakes studied (Odada and Olago, 2002). Interestingly, all clones retrieved from Antarctic lakes were specific to that region possibly explained by the perennial ice-cover acting as a physical barrier to dispersal. The lakes of the McMurdo Dry Valleys have been isolated from exchange with atmosphere by their ice covers for perhaps a 1,000 years (Lyons et al., 1998; Poreda et al., 2004). Similarly, the majority of African clones were also region-specific, except for 2 out of 43 clones, which were shared with lakes in Mongolia. In contrast, 22% of OTUs present in Mongolian lakes were also present in German samples. As expected, the ratio of shared OTUs was higher within each region.

According to the distance-decay hypothesis, community similarity declines with increasing geographic distance if species tend to be locally adapted or if they are dispersal limited (Nekola and White, 1999). If the patterns of community composition observed could be explained merely by geographical distance, lakes closer in space would be closer in community structure. African lakes are on average at a distance of 5,800 km from German lakes, whereas Mongolian lakes are located ~10,000 km away, and yet, Mongolian and German lakes are more closely related in community structure (Figure 1). If dispersal through air or by bird vectors could explain the pattern observed, we would expect that African and German lakes would be more similar because of geographic distance and/or bird seasonal migration. However, the pattern observed suggests instead a latitudinal explanation and, possibly, climate-related. Despite differences in climate between Germany and Mongolia, these lakes are subjected to more similar climatic regimes than lakes located in Africa and Antarctica (Lima-Ribeiro et al., 2015). Permutation tests (PERMANOVA) confirmed that both the geographic (Mongolian, German, African, Antarctic) and latitudinal (temperate, tropical, polar) region influence the observed community structure (*P* = 0.001). Nevertheless, we cannot discard that other regional factors based on the local conditions influence the AAP assemblages. For example, lakes in the two northern regions have similar age and have probably been exposed to similar major climatic events, namely the last glacial period of the Quaternary, ending ~11,700 years ago with the start of the Holocene.

**Figure 2:**
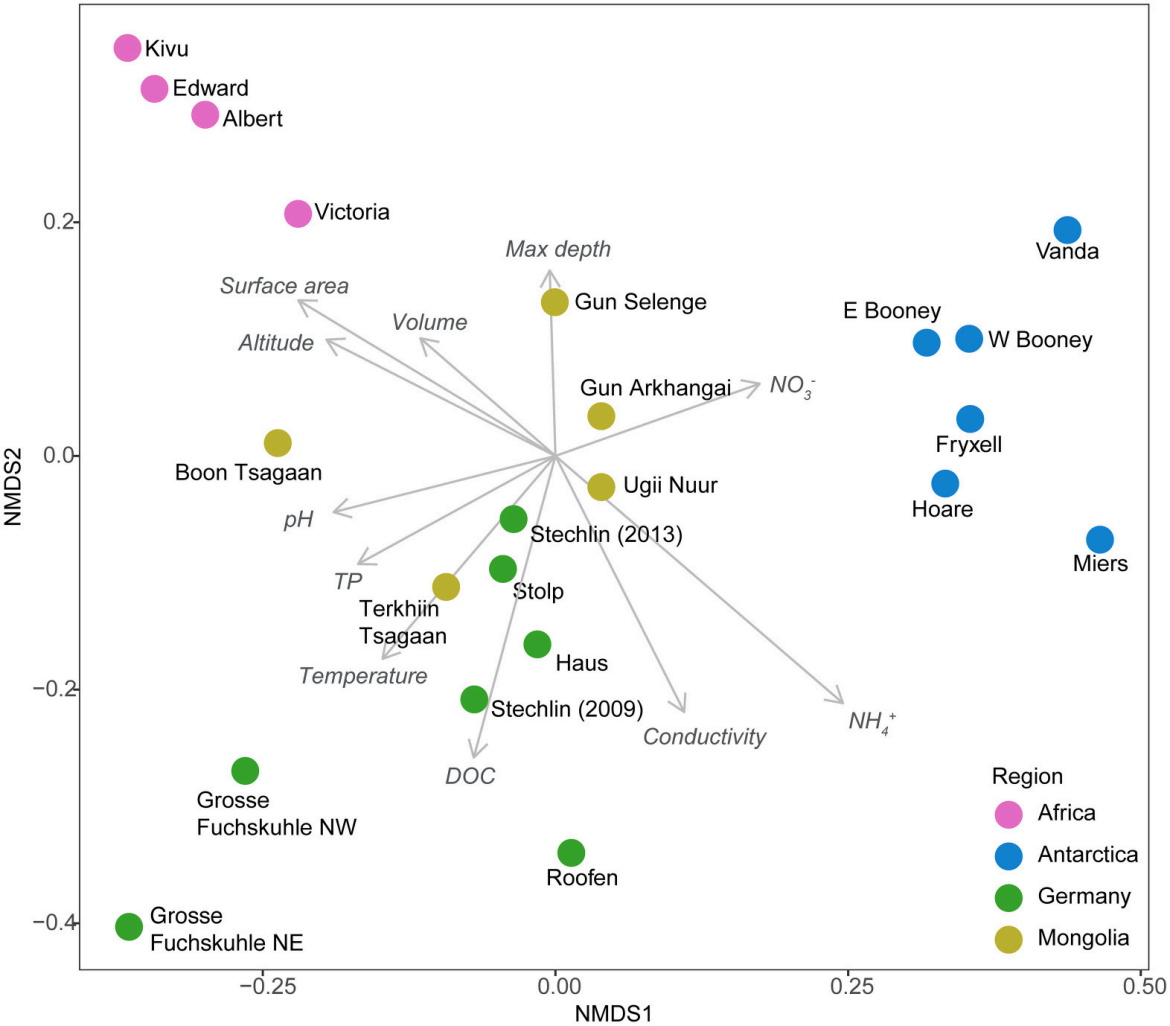
**Non-metric multidimensional scaling (NMDS) plot based on Bray–Curtis distances of OTU composition of AAP communities with vector overlays of the environmental factors (note that none of the variables were statistically significant)**. Colors indicate the different geographic regions studied. Sample from the SW basin Lake Grosse Fuchskuhle was excluded from the analysis for plotting purposes since it represents a very distant sample (see Figure 1). DOC, Dissolved organic carbon; TP, total phosphorous.

Additionally, a certain degree of influence of local conditions on the pattern of community structure was found (Mantel test, *R* = 0.30, *P* = 0.001). Linear fitting of environmental factors to the nMDS ordination revealed a certain effect of several measured variables, but none was statistically significant (*P* > 0.05; Figure 2). In contrast, lake trophy did not explain the assemblages (*P* > 0.05). Altogether, these results indicate that latitude and potentially climatic factors are strong determinants of AAP community composition, but the patterns observed reflect a certain adaptation to specific environmental features.

### Diversity of AAP bacteria

**Figure 3:**
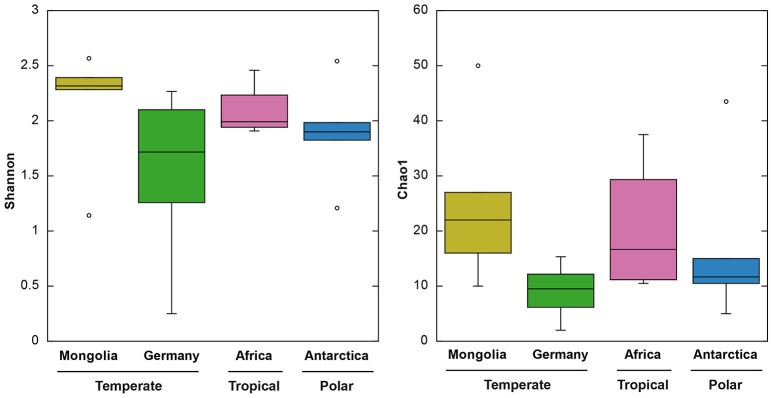
**Boxplots of Shannon (left) and Chao1 (right)** indices of the four different geographic regions. From top to bottom, the horizontal lines of the box represent the upper-quartile, median and lower-quartile. Whiskers extending from top and bottom of the box represent maximum and minimum values.

Sequence data from a total of 791 clones obtained in our study and 243 clones previously obtained from German lakes using the same procedures (Salka et al., 2011) were included in our analyses. Sequences resulted in 188 different OTUs, from which 172 (91%) were unique to a particular geographic region. The number of OTUs in each region ranged between 42 and 64, and within lakes the range was 3–23. Since OTU diversity estimates depend on the sequencing effort, we normalized the dataset for comparative purposes. Although a slightly higher richness (Chao1) and diversity (Shannon) were detected in the Mongolian lakes, we did not find significant differences depending on geographic region (ANOVA, *P* > 0.05, Figure 3). Likewise, no differences in Shannon and Chao1 indices were observed when grouping by latitudinal region (ANOVA, *P* > 0.05). While differences in species richness along latitudinal gradients are clear in macro-organisms with higher proportions of species in the tropics (Gaston, 2000), the trend is more ambiguous for microorganisms. For bacteria, both a significant richness-latitude relationship in marine biomes (Fuhrman et al., 2008) and a lack of latitudinal pattern of diversity in marine environments (Ghiglione et al., 2012) and soils (Fierer and Jackson, 2006) has been reported. Likewise, for inland waters the topic of microbial biogeography is currently greatly debated, but few data are available, except for mid latitudes (mostly temperate regions), making impossible a comprehensive latitudinal comparative study (Brendan Logue and Lindström, 2008; Romina Schiaffino et al., 2011; Sarmento, 2012). Although limited to a particular functional group, our study does not provide supporting evidence of a richness-latitude relationship in inland waters. These results may not be conclusive since diversity estimates are a function of the sequencing effort and we used cloning and sequencing instead of High-Throughput Sequencing (HTS). As a tradeoff, Sanger sequencing provides higher quality reads. We considered the Sanger platform more appropriate for this study (see below), yet we cannot assure that a deeper sequencing effort would provide the same view on diversity patterns. Although our dataset has some limitations, this is up to date the largest survey investigating AAP diversity patterns.

### Phylogeny of AAP bacteria

**Figure 4:**
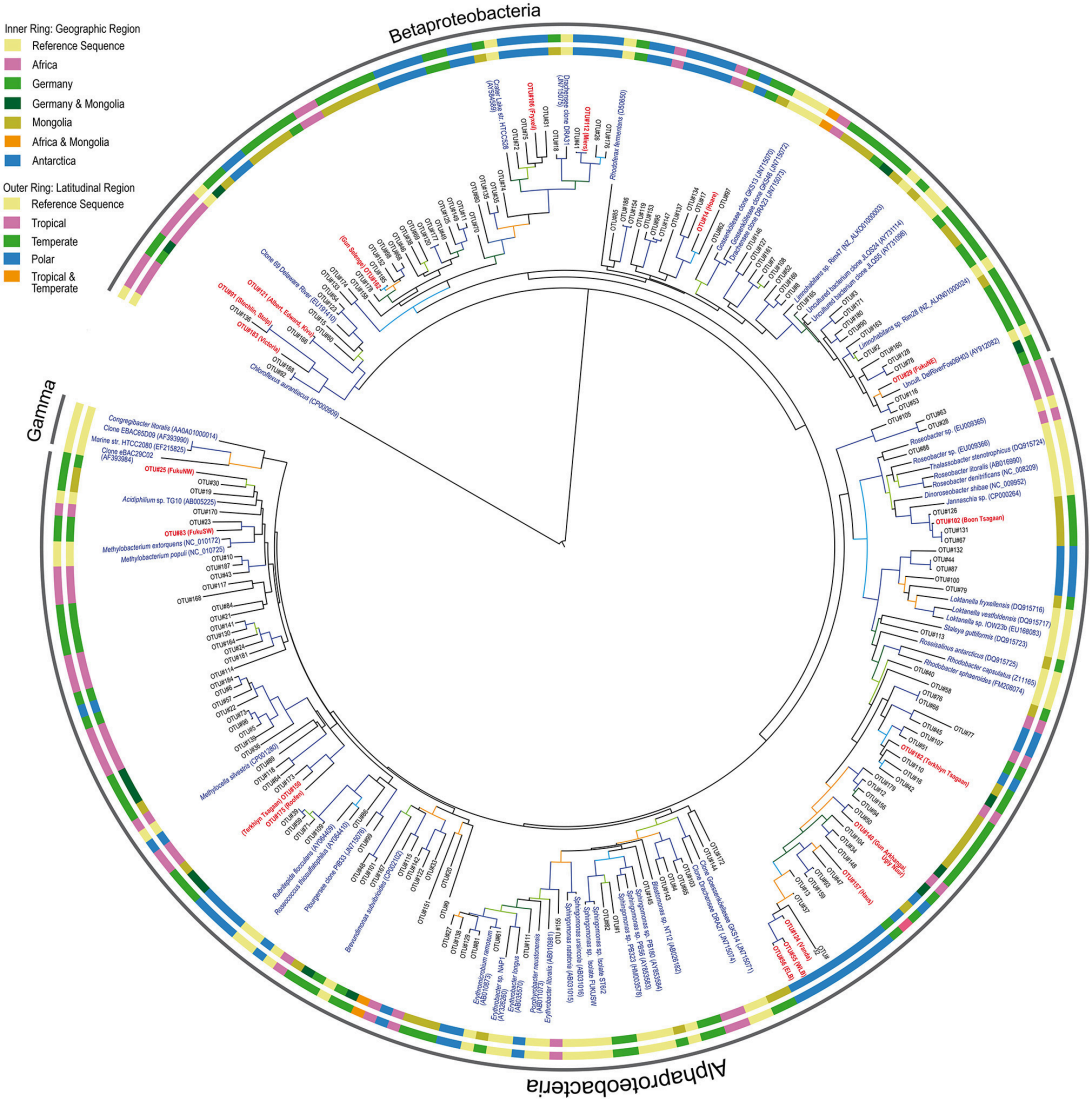
**Phylogenetic tree constructed from partial *puf*M gene sequences**. Each representative sequence of the clones grouped as OTUs (95%) is indicated as OTU number. The most abundant OTUs in each library are boldfaced in red and the corresponding lake indicated in parentheses. Reference sequences and their GenBank accession numbers are shown in blue. Geographic and latitudinal categories from each sequence are shown as inner and outer ring, respectively. The green sulfur bacterium *Chloroflexus aurantiacus* was set as outgroup. Tree branches are colored according to the bootstrap value (≥50: light green, >60: dark green, >70: orange, >80: light blue, >90: dark blue).

Studies on the taxonomy of freshwater AAPs are restricted to lakes and rivers of the Northern hemisphere (Waidner and Kirchman, 2008; Cottrell et al., 2010; Salka et al., 2011; Martinez-Garcia et al., 2012; Čuperová et al., 2013; Caliz and Casamayor, 2014). Here, we report for the first time sequences from previously unexplored regions and provide new information on how AAP communities differ taxonomically at global scales. Despite the potential of HTS to describe microbial diversity and the fact that we have previously used it for sequencing the *puf*M in marine samples (Ferrera et al., 2014), we decided to use cloning and Sanger sequencing because the quality and the length of Sanger sequences is better and these are critical to perform accurate phylogenetic analyses, particularly when few reference sequences are available. Since we were studying largely underexplored ecosystems, we believe that this was a better approach for the description and proper taxonomic classification of new freshwater AAP diversity. As observed in the phylogenetic tree (Figure 4), most of the sequences reported here are fairly distant from any reference sequence (81.8% average of similarity to best hit in Genbank database).

**Figure 5:**
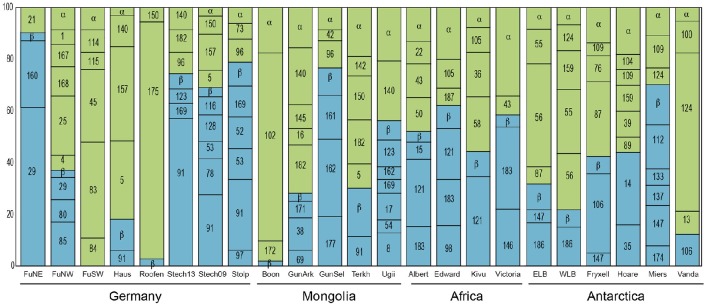
**Relative contribution of each OTU to the total diversity of the AAP community in the different lakes**. OTUs belonging to the *Alphaproteobacteria* are shown in green and to the *Betaproteobacteria* in blue. The name of the abundant OTUs is indicated in the histogram bars by its number. OTUs representing <5% of relative abundance in each sample have been grouped into other *Alphaproteobacteria* (α) or other *Betaproteobacteria* (β) to ease visualization. For phylogenetic placement of each OTU, see Figure 3. FuNE, Grosse Fuchskuhle NE; FuNW, Grosse Fuchskuhle NW; FuNW, Grosse Fuchskuhle NW; Stech13, Stechlin 2013; Stech09, Stechlin 2009; Boon, Boon Tsagaan; GunArk, Gun Arkhangai; GunSel, Gun Selenge; Terkh, Terkhiin Tsagaan; Ugii, Ugii Nuur; ELB, East Lake Bonney; WLB, West Lake Bonney.

Previous studies have shown that the phylogenetic composition of freshwater AAPs is composed mainly of members of the *Alpha*- and *Betaproteobacteria*, whereas the presence of *Gammaproteobacteria* in such ecosystems remains controversial (Caliz and Casamayor, 2014). Salka et al. (2011) found that German lakes harbored diverse members of the *Alpha*- and *Betaproteobacteria* and that their contribution varied largely depending on the lake. In high mountain lakes, two independent studies found contrasting results (Čuperová et al., 2013; Caliz and Casamayor, 2014). While the alpine Lake Gossenköllesee located in the Tyrolean Alps, Austria, was almost exclusively inhabited by *Sphingomonadales* species (*Alphaproteobacteria*; (Čuperová et al., 2013)), a survey of four Pyrenean lakes revealed that most *puf*M clones belonged to *Limnohabitans* (*Betaproteobacteria*; Caliz and Casamayor, 2014). We found highly variable AAP assemblages across lakes and regions (Figure 3). AAP communities were composed of different members of the *Alpha*- and *Betaproteobacteria*, generally at similar proportions (on average, 58 and 42% relatively), but with some exceptions in which either one dominated (Figure 5). Interestingly, we did not find gammaproteobacterial sequences, which are typically confined to marine ecosystems, estuaries and other inland saline environments such as saline Tibetan lakes, where they can dominate (Jiang et al., 2009, 2010; Ferrera et al., 2014).

At the phylotype level, our results suggest a strong macro-geographical signal in taxonomical composition, being clearly less variable across lakes within the same region than across regions (Figure 5). In fact, most phylotypes were more closely related to other phylotypes from the same geographic location (see Figure 4). In terms of abundance, the most abundant OTUs in each lake were generally different (Figure 5). Rank abundance profiles of the whole dataset did not show the common pattern of a few dominant phylotypes and many rare taxa (Liu et al., 2015), likely due to the lack of cosmopolitan phylotypes. Contrarily, we observed a clear niche partitioning of the different taxa. In African lakes, the most abundant phylotypes were affiliated to the *Betaproteobacteria*, in particular phylotypes belonging to an uncultured cluster, which includes the previously reported Clone 69 from Delaware River (EU191410). In addition, OTU #146 clustering with *Limnohabitans* was also fairly abundant in Lake Victoria.

In Antarctica, Lakes Bonney and Vanda were largely dominated by the alphaproteobacterial OTU#55, #56, and #124 closely related among them. Although these clones cluster within the *Rhodobacter* clade, they are highly dissimilar, likely representing new organisms. Contrarily, the most abundant phylotypes in Lakes Fryxell and Miers were OTU#106 and #112, respectively, both related to Crater Lake strain HTCC528 (AY584589), while *Rhodoferax*-related OTU#14 dominated Lake Hoare, all three belonging to the *Betaproteobacteria*. With the exception of Lake Gun Selenge in which the betaproteobacterial OTU#162 was the most abundant clone, Mongolian lakes were dominated by members of the *Alphaproteobacteria*, particularly OTU#140 and #182, within the *Rhodobacter* cluster but fairly distant, and OTU#102 (*Jannaschia*-like). As previously described by Salka et al. (2011), German lakes were dominated by either members of the *Alpha*- or *Betaproteobacteria* depending on the lake. However, in that publication the most frequent group was reported to be a cluster of sequences related to *Rhodoferax*. New phylogenetic placement of these sequences revealed that some of these are more closely related to *Limnohabitans* (OTU#29) or to a large cluster of betaproteobacterial sequences that represent an unidentified group (OTU#91).

Overall, this study represents the first attempt to study the AAP community ecology at global scales. Our results confirm that freshwater AAP members are restricted to the *Alpha*- and *Betaproteobacteria* regardless of the geographic region, while *Gammaproteobacteria* AAPs do not occur in fresh waters, supporting the hypothesis of taxonomic partitioning along salinity gradients (Jiang et al., 2009, 2010; Caliz and Casamayor, 2014). We cannot discard that a proportion of the diversity may have been missed due to primer biases or to limited sequencing depth. However, the fact that the vast majority of phylotypes showed only moderate relatedness to reference sequences reflects that the diversity of freshwater AAPs is largely undescribed and that many of the sequences reported here could represent new organisms.

## Conclusions

The detection of AAPs in all lakes surveyed confirms the widespread distribution of this functional group in contrasting freshwater ecosystems located across wide geographic and latitudinal gradients, including perennially ice-covered Antarctic lakes. In terms of abundance, no clear patterns are found across latitudes. Likewise, species richness and diversity do not strongly correlate with latitudinal gradients contrary to what has been observed for other organisms and biomes. Composition of AAPs is highly variable across lakes and regions but generally composed of members of the *Alpha*- and *Betaproteobacteria*, confirming the taxonomic partitioning of the *Gammaproteobacteria* to high salinity environments. Although most lakes seem to harbor idiosyncratic communities and the pattern of AAP community structure is modulated to a certain extent by the local environmental variables, a strong macro-geographical signal in taxonomical composition was found. Summarizing, our results show that the occurrence and distribution patterns of freshwater AAPs at a global scale are likely caused by a combination of small-scale environmental conditions (specific to each lake and local region) and large-scale geographic factors (climatic regions across a latitudinal gradients).

## Author contributions

IF and HG conceived this work. HG, HS, JP, and AC participated in the sampling campaigns and provided samples. IF performed all laboratory analyzes and JG helped with phylogenetic sequence reconstruction. IF analyzed the data and wrote the paper. All authors made comments and suggestions to the text.

### Conflict of interest statement

The authors declare that the research was conducted in the absence of any commercial or financial relationships that could be construed as a potential conflict of interest.
